# Heme Biosynthetic Gene Expression Analysis With dPCR in Erythropoietic Protoporphyria Patients

**DOI:** 10.3389/fphys.2022.886194

**Published:** 2022-07-18

**Authors:** Francesca Granata, Valentina Brancaleoni, Jasmin Barman-Aksözen, Margherita Scopetti, Giacomo De Luca, Silvia Fustinoni, Irene Motta, Elena Di Pierro, Giovanna Graziadei

**Affiliations:** ^1^ Fondazione IRCCS Ca’ Granda Ospedale Maggiore Policlinico, U.O.C. Medicina Generale, Milano, Italy; ^2^ Department of Medical Institutes, Institute of Laboratory Medicine, Stadtspital Zürich, Zürich, Switzerland; ^3^ Università degli Studi di Milano, Milan, Italy; ^4^ EPIGET—Epidemiology, Epigenetics, and Toxicology Lab, Department of Clinical Sciences and Community Health, Fondazione IRCCS Ca’ Granda Ospedale Maggiore Policlinico, U.O.S Tossicologia, Università degli Studi di Milano, Milan, Italy; ^5^ Department of Clinical Sciences and Community Health, Università degli Studi di Milano, Milan, Italy

**Keywords:** heme biosynthesis, porphyria, erythropoietic protoporphyria, digital PCR, ALAS2 gene

## Abstract

**Background:** The heme biosynthesis (HB) involves eight subsequent enzymatic steps. Erythropoietic protoporphyria (EPP) is caused by loss-of-function mutations in the ferrochelatase (FECH) gene, which in the last HB step inserts ferrous iron into protoporphyrin IX (PPIX) to form heme.

**Aim and method:** The aim of this work was to for the first time analyze the mRNA expression of all HB genes in peripheral blood samples of patients with EPP having the same genotype FECH c.[215dupT]; [315-48T > C] as compared to healthy controls by highly sensitive and specific digital PCR assays (dPCR).

**Results:** We confirmed a decreased FECH mRNA expression in patients with EPP. Further, we found increased ALAS2 and decreased ALAS1, CPOX, PPOX and HMBS mRNA expression in patients with EPP compared to healthy controls. ALAS2 correlated with FECH mRNA expression (EPP: *r* = 0.63, *p* = 0.03 and controls: *r* = 0.68, *p* = 0.02) and blood parameters like PPIX (EPP: *r* = 0.58 *p* = 0.06).

**Conclusion:** Our method is the first that accurately quantifies HB mRNA from blood samples with potential applications in the monitoring of treatment effects of mRNA modifying therapies *in vivo*, or investigation of the HB pathway and its regulation. However, our findings should be studied in separated blood cell fractions and on the enzymatic level.

## Introduction

Heme is an integral part of hemoproteins and essential for human life. Around 80% of the total body heme is produced during the erythropoiesis to synthesize hemoglobin and 15% in the liver to synthesize cytochrome P450 class enzymes. (Peoc’h et al., 2016; Phillips 2019). Heme is built in eight subsequent enzymatic steps, occurring either in the mitochondrion or the cytosol as comprehensively described in the current review article by Phillips (2019) and shortly summarized in the text box in the Supplementary Material. Mutations in each of the steps of the heme biosynthesis (HB) pathway cause a specific type of porphyria, which are rare intoxication-type inborn errors of metabolism characterized by the accumulation of specific heme precursors. While the enzymatic reactions to form heme are similar in all tissues, their regulation differs between the building sites (Chiabrando et al., 2014; Phillips 2019).

The first and rate limiting enzyme of the HB has a housekeeping isoform located in the mitochondrion, aminolevulinate synthase 1 (ALAS1), and an erythroid-specific isoform, ALAS2. The second rate-limiting enzyme is ferrochelatase (FECH) which inserts ferrous iron into PPIX. The expression of FECH increases during erythroid differentiation and is controlled by Sp1, BF-E2, and GATA1 transcription factors (Taketani et al., 1992; Tugores et al., 1994). Mutations causing a partial deficiency of FECH lead to erythropoietic protoporphyria (EPP) which is characterized by the accumulation of PPIX in the erythrocytes. The lead symptom of EPP is painful phototoxicity caused by the absorption of the energy from the visible light range by PPIX. Further, PPIX is hepatotoxic and in around 5% of patients with EPP leads to a potentially fatal liver failure (Wahlin et al., 2011; Wensink et al., 2021; Di Pierro et al., 2022). In addition, most patients are affected by disturbances in the iron metabolism like low hemoglobin, ferritin, transferrin saturation, and iron accompanied by microcytic, hypochromic anemia (Barman-Aksözen et al., 2015). Despite these indications for iron deficiency, earlier studies and case reports from patients with FECH deficient EPP demonstrated that administration of iron, while improving the anemia, also induced an increase in PPIX, phototoxicity and serum transaminases indicating induction of liver cell damage (Holme et al., 2007; Buzzetti et al., 2022). The observed increase in PPIX under iron substitution is likely mediated by stimulated erythropoietic porphyrin synthesis *via* induction of ALAS2 translation by iron. The ALAS2 mRNA carries an iron response element (IRE) in its 5′-UTR (untranslated region) which upon binding of iron response protein 2 (IRP2) inhibits translation in case iron is scarce (Pantopoulos 2004; Poli et al., 2021). Under iron-depleted conditions, IRP2 is degraded and ALAS2 mRNA can be translated. ALAS2 expression at the mRNA and protein level has been shown to be increased in peripheral blood samples of patients with FECH deficient EPP compared to healthy controls (Barman-Aksözen, et al., 2015) Gain-of-function mutations in ALAS2 lead to X-linked pprotoporphyria(XLP) associated with accumulation of PPIX during the erythropoiesis and a clinical presentation comparable to that of EPP (Whatley et al., 2008; Brancaleoni et al., 2016).

ThThis work aimedo analyze the mRNA expression levels of all the genes of the HB pathway in peripheral blood samples of patients affected by FECH deficient EPP as compared to healthy controls by highly sensitive and specific digital PCR assays (dPCR) (Granata et al., 2018; Quan et al., 2018; Feng et al., 2020). Therefore, 11 patients with EPP having the same genotype *FECH* c.[215dupT]; [315-48T > C] were compared to 11 healthy controls with the wild type genotype c.[315-48T = ]; [315-48T = ]. The loss-of-function mutation c.[215dupT] causes a frameshift with stop codon and has a 27,4% prevalence in the Italian EPP patient cohort (Ventura et al., 2020). FECH-deficient EPP is usually caused by loss-of-function mutations in FECH *in trans* to the splice modulating single nucleotide variant c. [315-48T > C] (Gouya et al., 2002). Both the wild type FECH c.[315-48T] and c.[315-48C] variants are associated with a partial aberrant splicing of the FECH mRNA, leading to nonsense-mediated decay caused by a premature stop codon in the retained intronic sequence which is more pronounced in the presence of the c.[315-48T > C] genotype (Rüfenacht et al., 1998; Kieke et al., 2019). Further, *in vitro* studies in the erythroleukemic cell line K562 and lymphoblastoid cell lines derived from patients with EPP and healthy controls showed a dose-dependent increase in aberrant FECH mRNA splicing under iron-depleted cell culture conditions that is more pronounced in the more common c.[315-48T] genotype (Barman-Aksözen et al., 2013). Around 90% of the Caucasian population carry the FECH c.[315-48T] variant, and the 11 healthy controls selected for this study were all confirmed to have this more prevalent genotype (Gouya et al., 2006).

## Methods and Materials

### Study Subjects

Eleven EPP patients referred to the Rare Disease Centre at Fondazione Ca’ Granda Policlinico of Milan and eleven age-matched control individuals were included. Freshly drawn full blood samples were collected in EDTA tubes and immediately processed. The erythrocyte protoporphyrins, i.e., PPIX and Zinc protoporphyrin (ZnPP), concentrations were determined as routine analysis, as previously described (Brancaleoni et al., 2018) Table 1 reports serum iron, hemoglobin (Hb) and mean corpuscular Hb (MCH) data retrieved from the patient’s routine clinical records. The study was conducted in accordance by the Declaration of Helsinki for medical research. All subjects involved in this study signed informed consent for the diagnosis and research approved by the ethics committee of our institution and the identities of the study participants were anonymized.

**TABLE 1:** Clinical and biochemical findings: PPIX [reference range: 0.0–3.0 µg/gHb] with percentage of PPIX [85—100%] and ZnPP [0—15%]; serum iron with ranges for female [37–145 μg/dl] and male [59–158 μg/dl]; MCH for female [25–35 pg] and male [25–35 pg]; Plasma peak and FECH genotype (Pt = patients; Ct = controls; NR = not reported in clinical folder; ND Not detected).

Pt	Age	Sex	Plasma Peak nm	PPIX (µg/gHb)	% PPIX	% ZnPP	Serum iron (µg/dl)	Hb G/dL	MCH (pg)	FECH Analysis
Pt1	29	F	632	68	92	8	19	10,4	22,9	c.[215dupT]; [315-48T > C]
Pt2	30	M	634	153	98	2	51	13,4	26,7	c.[215dupT]; [315-48T > C]
Pt3	32	F	634	86	96	4	24	11,4	23,8	c.[215dupT]; [315-48T > C]
Pt4	44	M	634	63	NR	NR	NR	NR	NR	c.[215dupT]; [315-48T > C]
Pt5	44	F	633	NR	NR	NR	60	11,7	26,7	c.[215dupT]; [315-48T > C]
Pt6	21	M	634	38	94	6	155	15	29,3	c.[215dupT]; [315-48T > C]
Pt7	24	F	633	30	9	9	49	13,4	30,2	c.[215dupT]; [315-48T > C]
Pt8	36	M	635	100	98	2	66	14,4	26,9	c.[215dupT]; [315-48T > C]
Pt9	46	F	634	30	93	7	77	12,3	26,1	c.[215dupT]; [315-48T > C]
Pt10	36	M	632	135	98	2	69	13,2	25,2	c.[215dupT]; [315-48T > C]
Pt11	50	M	631	152	98	2	130	14,8	29,4	c.[215dupT]; [315-48T > C]
Ct1	36	F	ND	ND	ND	ND	ND	ND	ND	c.[315-48T = ]; [315-48T = ]
Ct2	32	M	ND	ND	ND	ND	ND	ND	ND	c.[315-48T = ]; [315-48T = ]
Ct3	42	M	ND	ND	ND	ND	ND	ND	ND	c.[315-48T = ]; [315-48T = ]
Ct4	33	F	ND	ND	ND	ND	ND	ND	ND	c.[315-48T = ]; [315-48T = ]
Ct5	51	F	ND	ND	ND	ND	ND	ND	ND	c.[315-48T = ]; [315-48T = ]
Ct6	29	M	ND	ND	ND	ND	ND	ND	ND	c.[315-48T = ]; [315-48T = ]
Ct7	16	M	ND	ND	ND	ND	ND	ND	ND	c.[315-48T = ]; [315-48T = ]
Ct8	48	F	ND	ND	ND	ND	ND	ND	ND	c.[315-48T = ]; [315-48T = ]
Ct9	50	M	ND	ND	ND	ND	ND	ND	ND	c.[315-48T = ]; [315-48T = ]
Ct10	47	F	ND	ND	ND	ND	ND	ND	ND	c.[315-48T = ]; [315-48T = ]
Ct11	28	F	ND	ND	ND	ND	ND	ND	ND	c.[315-48T = ]; [315-48T = ]

### RNA Isolation, Reverse Transcription, and Digital PCR

Total mRNA was extracted from leukocytes with a Maxwell 16 automated extractor (Promega Corporation, Madison, WI, United Sates) according to the manufacturer’s protocol with slight modifications, as previously described (Fiorentino et al., 2016).

One hundred ng of total RNA was reverse transcribed (RT-PCR), using the ViLo Master Mix (Thermo Fisher Corporation Inc., San Francisco, CA, United Sates). Heme biosynthetic gene expression was assessed by dPCR on a Quantstudio^®^ 3D Digital PCR System (Thermo Fisher Corporation Inc., San Francisco, CA United Sates), using specific and differentially labeled Gene Expression TaqMan^®^ probe mixes, to perform dPCR experiments (Table 2).

**TABLE 2:** Gene expression TaqMan^®^ probes used in the study.

Gene	Probe
ALAS1	Hs00963537_m1-VIC
ALAS2	Hs00163601_m1-FAM
ALAD	Hs00765604_m1-VIC
HMBS	Hs00609296_g1-FAM
UROS	Hs03405152_m1-VIC
UROD	Hs01099757_g1-VIC
CPOX	Hs01071019_m1-FAM
PPOX	Hs00609392_m1-FAM
FECH	Hs01555261_m1-FAM
GUSb	Hs00939627_m1-VIC

Different amounts of total RNA were loaded onto the 3D Digital PCR Chip, using the QuantStudio^®^3D Digital PCR Chip Loader in a reaction mixture, consisting of the 2 × Quantstudio^®^3D Digital PCR Master Mix and 1× of each of the Gene Expression TaqMan^®^ probes. The chips were sealed and loaded onto the ProFlex™2xFlat PCR System (Thermo Fisher Corporation Inc., San Francisco, CA, United Sates) and cycled according to the following parameters: initial denaturation at 96°C for 10 min, followed by 40 or 45 cycles of amplification as follows, denaturation at 98°C for 30 s, annealing at 60 or 56°C for 2 min, and a final extension at 60°C for 2 min. The number of cycles and annealing temperatures were specific for each probe mix pairs used in each experiment.

After cycling, the end-point fluorescence of the partitions on the chips was measured by transferring the chips to the QuantStudio^®^3D chip reader; secondary analysis was performed with QuantStudio^®^3D Analysis Suite cloud software (application version 3.1.6-PRC-build18; algorithm version 4.4.10).

### Statistical Analysis

All raw data were divided or multiplied to consider 2.5 ng of mRNA for all experiments. The calculations are shown in Table 1 of the Supplementary Data S1. The normality tests were assessed, all data of copies/µl was transformed in logarithmic scale 10 and data were analyzed using an unpaired parametric *t*-test; nominal statistical significance was set at *p* < 0.05. The correlations were evaluated using Pearson’s correlation coefficient. All statistical analyses were performed using the GraphPad Prism software (version 9.0).

## Results

### Characteristics of Patients and Controls

The clinical and biochemical findings for patients and controls involved in the study are summarized in Table 1. Five females (34 ± 9 years) and six males (36 ± 10 years) with EPP were included in the study. The controls, i.e., six females (37 ± 11 years) and five males (38 ± 12 years), were not affected by EPP (healthy controls).

The EPP subjects exhibited typical plasma fluorescence peaks at 631–635 nm. The controls were negative for plasma fluorescence. The mean of erythrocyte PPIX was 85,6 ± 45 [normal range: 0–3 μg/g Hb], with the percentage of PPIX/ZnPP increased in all patients. All patients shared the same FECH mutation, i.e., c.[215dupT] *in trans* to the c.[315-48C] allele. All controls were healthy Caucasians with the more common FECH c.[315-48T] variant genotype for the FECH gene in homozygosity. We chose the same genotype on purpose to minimize all possible expression variability due to different genetic backgrounds (Table 1). The mean serum iron was 45.8 ± 24.4 [37–145 μg/dl] for female and 94.2 ± 45.4 [59–158 μg/dl] for male patients. The mean Hb in the females was 12,7 ± 1,6 [12–16 g/dl] and in the males 12,3 ± 1,3 [13.5–17.5 g/dl]. The MCH for the female patients shows a mean of 26,5 ± 2,4 [25–35 pg] and for the male patients 27,1 ± 2,1 [25–35 pg].

### Digital PCR Conditions

The raw data used for the results and statistical analyses (mean; SD, Min and Max) are reported in the Supplementary Table S1. The experiments have been performed starting from mRNA reverse transcribed to cDNA at a concentration of 5 ng/μL. The dPCR experiments were set up with a gradient system of mRNA concentration, temperature and PCR cycles. Table 3 shows the final condition for all paired probes. The optimal amounts of cDNA ranged from 0.70 to 5 ng of total mRNA, depending on the endogenous expression of the respective mRNA. The amplification temperature ranged between 56 and 60°C and the number of cycles varied between 40X, predominantly associated with the highest temperature (60°C), and 45X associated with 56°C. All experiment setups showed a good quality of chip loading with a precision of less than 10% and the raw data was standardized to 2.5 ng of total mRNA to perform the statistical analysis (Supplementary Table S1).

**TABLE 3:** dPCR conditions for all genes.

FAM	VIC	Temperature (°C)	*n* of Cycle	Total ng
ALAS2	GUSb	60	40X	0.70
HMBS	ALAS1	56	45X	5
CPOX	UROS	56	45X	5
PPOX	UROD	60	40X	2.5
FECH	ALAD	56	45X	2.5

### Lower Expressed EPP Biosynthetic Pathway Genes Compared to CTRL

**FIGURE 1:**
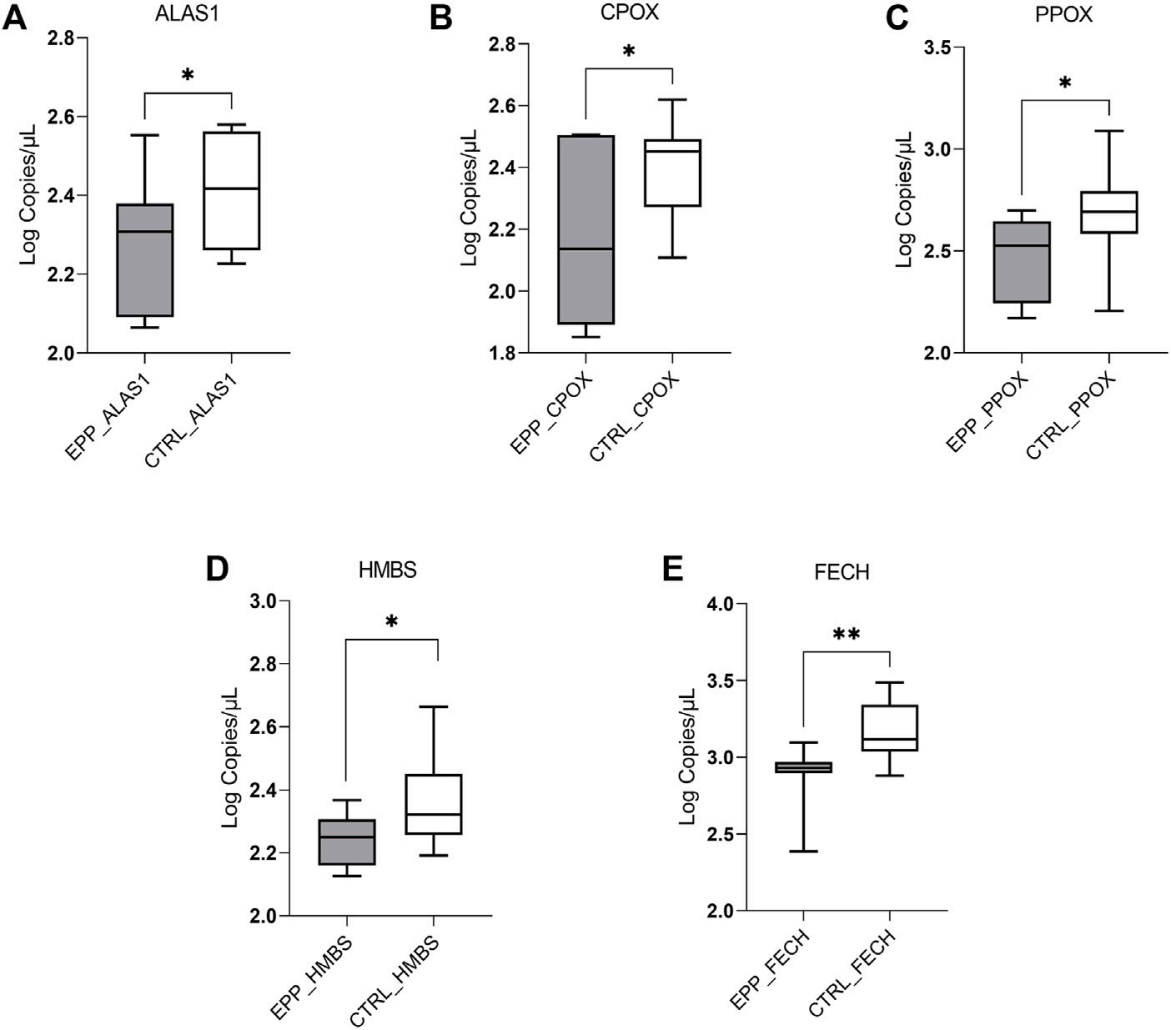
Lower EPP gene expression reported in Log of copies/µl for **(A)** ALAS1; **(B)** CPOX; **(C)** PPOX **(D)** HMBS **(E)** FECH genes, between EPP patients and Controls (p* < 0.05 **p ≤ 0.01).

The dPCR analysis showed a decreased quantity of mRNA of the three genes coding for enzymes located in the mitochondria: ALAS1 (EPP 198 ± 80.4 copies/µl; CTRL 273 ± 82.7 copies/µl *p* < 0.03); CPOX (EPP 191.4 ± 107.5 copies/µl; CTRL 265.5 ± 81 copies/µl *p* < 0.05) and PPOX (EPP 313.2 ± 129.4 copies/µl; CTRL 536 ± 300.5 copies/µl *p* < 0.05) (Figures 1A–C). Further, the mRNA of the cytosolic enzyme HMBS shows different means between both populations (EPP 174 ± 32.8 copies/µL; CTRL 245.3 ± 88.8 copies/µl *p* < 0.01) (Figure 1D). Finally, as it can be seen in Figure 1E, the FECH gene is lower expressed in the cohort of EPP patients (822.1 ± 248.5 copies/µl) compared to the controls (1543.3 ± 718 copies/µlp <0.003), confirming the effect of the mutation on this gene, i.e., a premature termination codon at a position that leads to the degradation of the FECH mRNA derived from this allele *via* the nonsense-mediated decay mechanism.

### ALAS2 and the Correlation With Hematological Parameters

**FIGURE 2:**
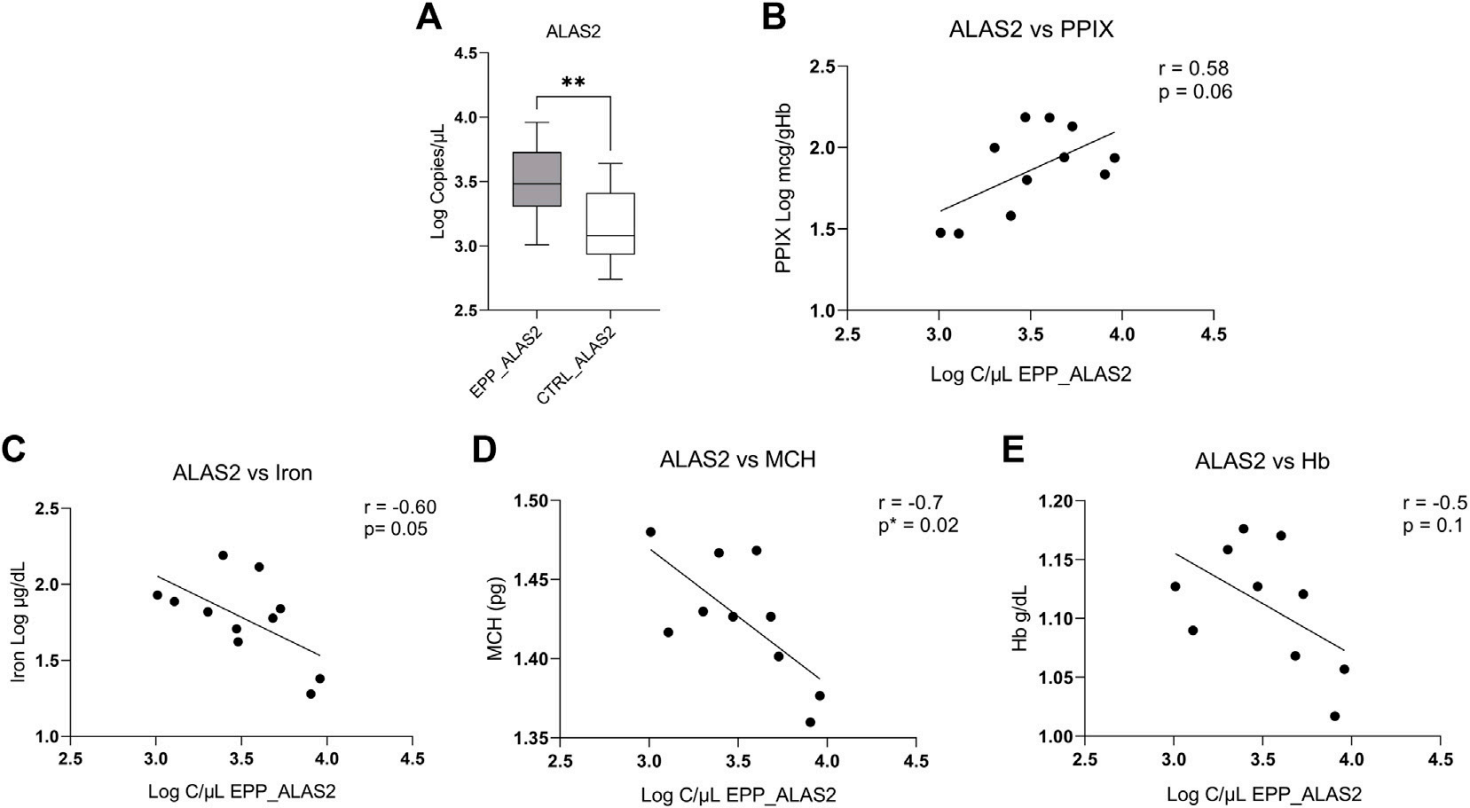
**(A)** Highest EPP gene expression reported in Log of copies/µL for ALAS2 **(B)** Positive tendency between total PPIX level of patients and copies/ul of ALAS2. **(C)** Negative correlation between the iron level of patients and copies/ul of ALAS2. **(D)** Negative and significative correlation between ALAS2 quantity and MCH (p* < 0.05; **p ≤ 0.01). **(E)** Negative tendency between ALAS2 and Hb.

Our measurements show a higher absolute expression of ALAS2 mRNA in patients with EPP (Figure 2A) (4013.5 ± 2638.1 copies/µl) as compared to the controls (1739.8 ± 1193 copies/µl *p* < 0.009). Figure 2B shows a positive tendency of ALAS2 with PPIX (*r* = 0.58 *p* = 0.06), but not between FECH and PPIX (correlation of gene expression *vs*. PPIX is reported in the Supplementary Figure S1). Our results show a negative inverse tendency between ALAS2 mRNA expression and iron (r = −0.60 *p* = 0.05) (Figure 2C) and MCH (r = −0.8 *p* = 0.02) (Figure 2D). Unfortunately, there was no correlation between ALAS2 mRNA and hemoglobin (r = -0.5 *p* = 0.1) (Figure 2E). In Supplementary Figure S2 we report the correlations with the haematological parameters and the Loc c/ul of FECH.

### Correlation Between ALAS2, ALAS1 and FECH

**FIGURE 3:**
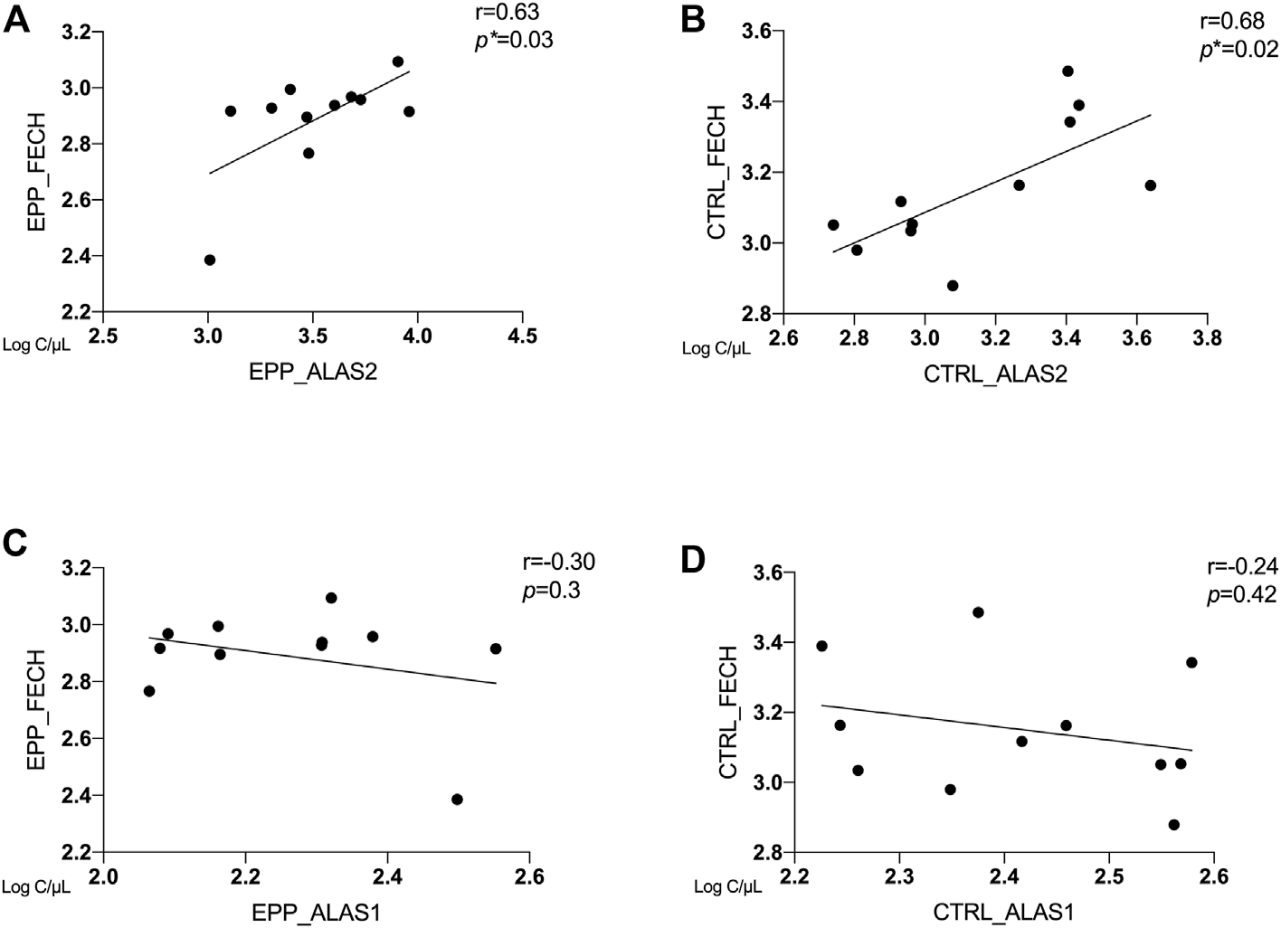
Correlation between ALAS2, ALAS1 and FECH between EPP and CTRL for **(A)** EPP_ALAS2 *vs*. EPP_FECH; **(B)** CTRL_ALAS2 *vs*. CTRL_FECH; **(C)** EPP_ALAS1 *vs*. EPP_FECH **(D)** CTRL_ALAS2 *vs*. CTRL_FECH. (p* < 0.05)

In Figures 3A–D, the correlations between copies/µl of FECH mRNA and the mRNAs of the rate-limiting enzymes ALAS1 (ubiquitous isoform) and ALAS2 (erythroid isoform) are given. The patients and the controls show a positive correlation between FECH and ALAS2 mRNA (EPP: Figure 3A *r* = 0.63 *p* = 0.03 and CTRL: Figure 3B *r* = 0.68; *p* = 0.02). In contrast, no correlation could be detected for FECH vs ALAS1 for both groups (Figure 3C *r* = −0.3; *p* = 0.3; Figure 3D *r* = −0.24; *p* = 0.42). Moreover, Supplementary Table S2 show no correlation between ALAS2 vs. other genes in EPP patients and CTRL.

### The Expression of Other Biosynthetic Pathway Genes in EPP

**FIGURE 4:**
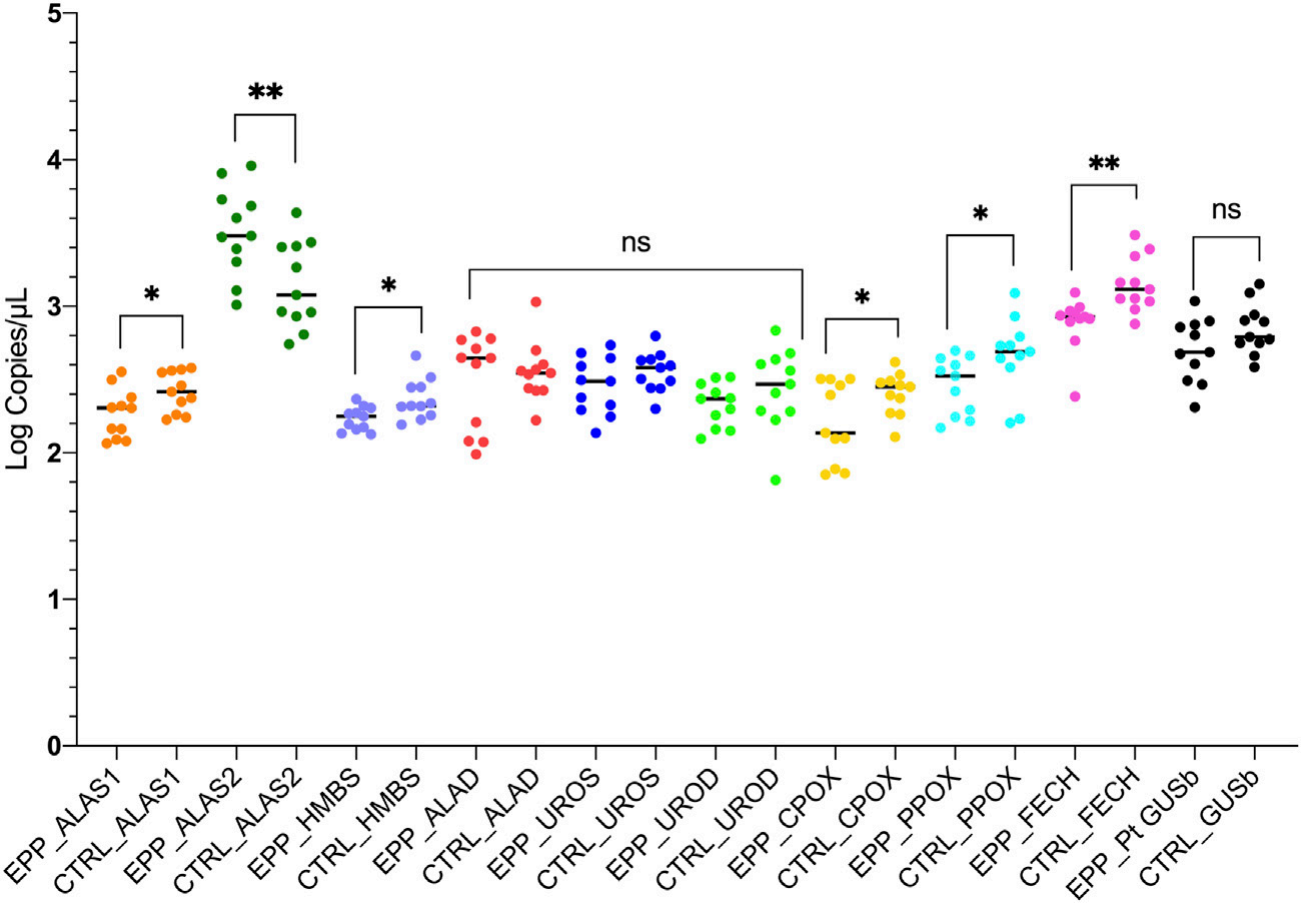
The graph reports the results of the experiments for both patients and controls in accordance to the heme biosynthetic pathway. Statistically significances are reported and each gene is labeled with a different color. (p* < 0.05 **p ≤ 0.01; ns = not significant).

Other gene expression analyses showed a similar mean and standard deviation (SD) of copies/µl between patients with EPP and CTRL. The results for the expression analyses of the ALAD, UROS and UROD genes (all cytosolic enzymes), were ALAD: EPP 379.4 ± 216.5 copies/µl; CTRL 397.5 ± 239.1 copies/µl *p* = 0.58; UROS: EPP 313.6 ± 135.2 copies/µl; CTRL 373.3 ± 116 copies/µl *p* = 0.19 and UROD: EPP 224.3 ± 73 copies/µl; CTRL 321.2 ± 174.8 copies/µl *p* = 0.27 (Figure 3 Supplementary Data SA–C). The housekeeping glucuronidase beta gene (GUSb) results are reported in Figure 3D in Supplementary Data S1 and showed no difference between EPP 561.3 ± 245.2 and CTRL 720.3.5 ± 233.4 (*p* = 0.1). A summary of all gene expression results is reported in Figure 4. The genes at the beginning and the end of the heme synthesis pathway show altered expressions, while genes in the middle of the pathway did not show differences between samples from patients and controls. The raw data for single patients and control are shown in Supplementary Figure S4.

## Discussion

Our study for the first time introduces the highly sensitive and specific quantification of all mRNAs in the HB pathway by dPCR assays. We analyzed mRNA extracted from buffy coats from peripheral blood samples of 11 patients with EPP having the same genetic background, i.e., the mutation FECH c.[215dupT]; [315-48T > C], and 11 healthy controls. As expected from a frameshift with stop codon mutation in trans to an allelic variant that is associated with partial aberrant splicing, the analysis showed an overall decreased expression of *FECH* mRNA by more than 50% in the EPP cohort (Figure 1E) compared to the control samples, which confirms the validity of the method already demonstrated in previous works and in other mutations of *FECH* gene (Brancaleoni, et al., 2018). Moreover, in paragraph 4 of Supplementary Data S1, we report the results of comparison of our cohort of 11 patients with 12 patients with other 9 mutations collected in previous experiment.

Interestingly, our results further show that the expression of the three mitochondrial enzymes ALAS1, CPOX, and PPOX and the cytosolic HMBS is lower in patients with EPP compared to healthy controls (Figures 1A–D). However, the findings need to be confirmed in more extensive studies, for example in blood cell fractions (e.g., erythroblasts), and with a more heterogeneous group of subjects. In addition, the observed alterations in the mRNA expression should be further investigated at the protein and enzyme activity levels to understand their biological relevance.

Another aspect emerging from our experiments is the vast increase in ALAS2 mRNA in the patient samples compared to the healthy controls, which confirms earlier findings in peripheral blood samples of patients with EPP with different genetic backgrounds conducted by RT-PCR (Barman-Aksözen, et al., 2015). Further, the results of the ALAS2 expression in our study correlate to biochemical parameters from the clinical records measured as routine diagnostic tests in the hospital (Table 1).

The negative tendency between MCH and iron with ALAS2 mRNA in patients could indicate a direct role of ALAS2 mRNA, specifically in the anemic condition of EPP (Figures 2C–E). The same was not true when comparing MCH and iron with the FECH mRNA expression (Supplementary Figure S2). At the same time, ALAS2 expression shows a positive tendency with PPIX, which supports and strengthens the hypothesis of an involvement of ALAS2 in worsening EPP clinical symptoms. A higher PPIX accumulation can exert a higher toxic effect on red blood cells and other EPP-affected districts, like the liver. A possible PPIX toxic effect on the red blood cell has been already suggested in previous work (Graziadei et al., 2022). None of the other HB gene expressions shows a significant correlation with PPIX, including FECH, the causative gene of EPP; their expression does not seem to affect the accumulation level of PPIX (Supplementary Fgure S1).

**FIGURE 5:**
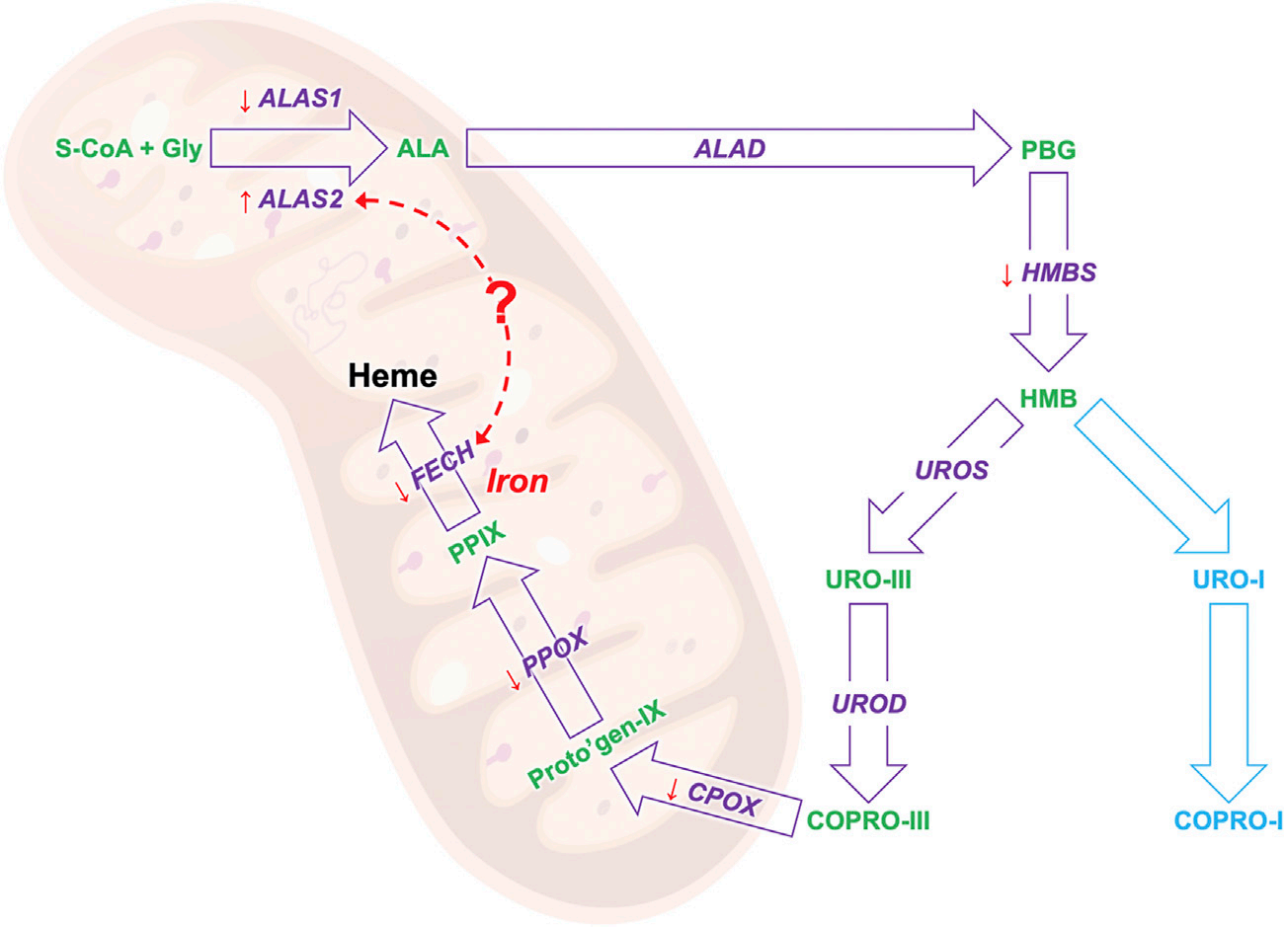
The results of the biosynthesis pathway mRNA study: the gene that encodes for the enzyme in given in purple and the heme precursors are given in greens. The orange arrows show the gene expression trend of EPP patients as compared to healthy controls, an arrow pointing down indicates a lower expression and up a high expression. The red dotted arrow indicates a possible link between the mRNA expression of the ALAS and FECH genes.

It has been shown in *in-vitro* experiments in HEPG2 cells that induced accumulation of PPIX diminishes respiratory control in the mitochondria and leads to the upregulation of dynamin-related protein (DRP-1) encoded by the DNML1 gene that mediates mitochondrial fission (Lin et al., 2013) suggesting a direct regulatory role of PPIX on mitochondrial gene expression. Moreover, our study confirms previous findings in patients with EPP and in the erythroleukemic cell line K562 showing that ALAS2 and FECH mRNA expression positively correlates both in EPP and CTRL (Figures 3A,B) (Barman-Aksözen et al., 2019). Both ALAS2 and FECH genes are regulated by the transcription factor GATA1. Although in another disease, Scherzer et al. found that the mRNA expression of both ALAS2 and FECH correlated to GATA1 expression regulated alpha-synuclein (SNCA) expression. This could suggest a direct link between ALAS2 and FECH expression in the heme synthesis (Figure 5) (Scherzer et al., 2008). Furthermore, their finding was confirmed by Santiago and Potashkin (2017) in a blood transcriptomic meta-analysis, who discussed a co-expression network and regulation of the heme biosynthesis genes (Santiago and Potashkin 2017). As transcriptional and post-transcriptional mechanisms can also affect the function of these genes, further studies on protein could be performed in the future, to check for a possible correlation between expression and protein quantification.

Overall, our findings in a fixed genetic background group could support the hypothesis that the overexpression of ALAS2 mRNA in EPP patients likely contributes to the disease etiology and therefore is a potential target for developing new treatment options in EPP (Ducamp et al., 2021; Halloy et al., 2021).

The presented method to accurately measure mRNA expression of the entire HB pathway in peripheral blood samples can be applied to study gene expression levels of the entire HB pathway, i.e., whether they are correctly maintained and regulated (Neeleman et al., 2018; Honor et al., 2021). Furthermore, the method can be used to monitor the *in vivo* expression patterns in patients under mRNA altering therapies like gene specific silencing RNAs (for example givosiran against ALAS1 in the acute hepatic porphyrias) or splice modulating oligos directed against FECH c.[315-48C] (Oustric et al., 2014; Halloy et al., 2020).

In conclusion, with this work, we set up a very sensitive and specific approach to study the mRNA expression of all genes of the HB pathway in blood samples. Our results confirm the low mRNA expression of FECH and the high ALAS2 expression in patients with EPP. Moreover, we showed low expression of other genes, particularly for the mitochondrial HB genes, which may pave the way for new insights into the functions and pathophysiological implications of this cellular organelle in EPP (Figure 5). By applying our method, we confirmed and accurately measured the correlation in the expression of two essential genes, FECH and ALAS2. At the same time, we also demonstrated correlations with blood parameters that will support the understanding of the underlying pathophysiologic mechanisms of EPP, such as the anemic condition found in most patients.

## Data Availability

The original contributions presented in the study are included in the article/Supplementary Material, further inquiries can be directed to the corresponding authors.
