# Supplementary material for: Heme Biosynthetic Gene Expression Analysis With dPCR in Erythropoietic Protoporphyria Patients
Source: Front Physiol. 2022 Jul 18;13:886194. doi: 10.3389/fphys.2022.886194 (PMC9340544; doi:10.3389/fphys.2022.886194)
Supplement: Supplementary file 1 [file DataSheet1.pdf]

Supplementary Material

1 Text box

The Heme biosynthesis

Two molecules ALA are converted into the monopyrrol porphobilinogen (PBG) by delta-aminolevulinate dehydratase (ALAD) in the cytosol. Hydroxymethylbilane synthase (HMBS) transforms four PBG molecules into the linear tetrapyrrole hydroxymethylbilane. Subsequently, uroporphyrinogen III synthase (UROS) forms the circular tetrapyrrole uroporphyrinogen III. In a multi-step reaction, uroporphyrinogen III is then converted to coproporphyrinogen III by the enzyme uroporphyrinogen III decarboxylase (UROD). The coproporphyrinogen III is transported back into the inter-membrane space of the mitochondria, where the enzyme coproporphyrinogen III oxidase (CPOX) forms protoporphyrinogen IX. Protoporphyrinogen IX is then oxidized to protoporphyrin IX (PPIX) by the enzyme protoporphyrinogen oxidase (PPOX). Finally, the enzyme ferrochelatase (FECH) inserts ferrous iron into protoporphyrin IX (PPIX) to form heme.

2 Supplementary Tables

Sample	ALAS1 [5 ng/µL]					ALAS 2 [0,70 ng/µL]				
	Raw C/µL	DF (2)	Precision	DP	DP above QT	Raw C/µL	MF (3,75)	Precision	DP	DP above QT
Pt_1	418,6	209,3	2.88%	19569	18005	2257,0	8060,7	1,86%	19071	17882
Pt_2	291,8	145,9	3,41%	18997	17403	830,4	2965,7	2,19%	19381	18441
Pt_3	713,6	356,8	2,32%	19373	17047	2548,9	9103,2	1,90%	19437	18233
Pt_4	232,0	116,0	3.65%	19487	18725	847,7	3027,6	2,25%	19336	16617
Pt_5	246,5	123,2	3,63%	18978	17422	1353,5	4833,9	1,93%	19238	18344
Pt_6	290,2	145,1	3.42%	19637	17509	690,9	2467,5	2,35%	18533	17526
Pt_7	629,8	314,9	2,57%	19634	16967	286,2	1022,1	3,46%	18303	17089
Pt_8	405,8	202,9	3,06%	19469	16312	564,4	2015,6	2,69%	18630	17774
Pt_9	240,3	120,1	3.63%	19299	18343	359,8	1284,8	2,86%	19130	18075
Pt_10	478,9	239,4	2,79%	19106	16041	1498,5	5351,8	1,94%	19083	17312
Pt_11	406,4	203,2	2.90%	19657	18051	1124,4	4015,7	2,04%	19175	17752
Mean	395,8	197,9	2,96%	19382	17439	1123,8	4013,5	2,32%	19029	17731
SD	160,9	80,4	0,50%	255	823	738,7	2638,1	0,50%	374	561
Min	232,0	116,0	2,32%	18978	16041	286,2	1022,1	1,86%	18303	16617
Max	713,6	356,8	3,63%	19657	18725	2548,9	9103,2	3,46%	19437	18441

CTRL_1	575,4	287,7	2,60%	19392	16165	1219,6	4355,7	1,90%	18879	16815
CTRL_2	336,7	168,3	3,21%	19485	17391	765,0	2732,0	2,32%	18630	17700
CTRL_3	758,2	379,1	2.28%	19375	18078	721,6	2577,1	1,89%	18767	17542
CTRL_4	350,4	175,2	3,18%	19585	17174	517,8	1849,1	2,63%	18702	17974
CTRL_5	729,5	364,7	2.35%	18711	17458	335,7	1199,0	3,17%	18520	17857
CTRL_6	708,2	354,1	2,52%	18898	15536	154,2	550,8	3,76%	19513	18453
CTRL_7	474,6	237,3	2.35%	19182	17287	711,7	2541,7	2,37%	18581	17487
CTRL_8	446,3	223,1	2,77%	19723	18374	179,9	642,6	4,20%	18685	17980
CTRL_9	740,1	370,0	2,33%	19737	17423	258,0	921,5	3,50%	19376	18494
CTRL_10	364,4	182,2	3,45%	18554	16905	255,5	912,4	3,54%	19237	18200
CTRL_11	522,1	261,0	2,62%	19549	18035	239,6	855,9	3,65%	19308	18252
Mean	546,0	273,0	2,84%	19290	17257	487,1	1739,8	2,99%	18927	17887
SD	165,6	82,8	0,40%	405	831	334,0	1192,9	0,80%	360	490
Min	336,7	168,3	2,33%	18554	15536	154,2	550,8	1,89%	18520	16815
Max	758,2	379,1	3,45%	19737	18374	1219,6	4355,7	4,20%	19513	18494

Sample	ALAD [2,5 ng/μL]					HMBS [5 ng/μL]				
	Raw C/μL	DF (0)	Precision	DP	DP above QT	Raw C/μL	DF (2)	Precision	DP	DP above QT
Pt_1	671,61		3,17%	19177	17847	466,24	233,12	2.75%	19569	Pt_1
Pt_2	602,24		3,29%	19477	18238	418,56	209,28	2,92%	18997	Pt_2
Pt_3	446,43		2,82%	19096	17608	369,56	184,78	3,01%	19373	Pt_3
Pt_4	120,25		5,11%	19570	17979	289,18	144,59	3.30%	19487	Pt_4
Pt_5	161,76		4,38%	19465	18239	271,14	135,57	3,47%	18978	Pt_5
Pt_6	97,675		5,63%	19869	18070	312,32	156,16	3.30%	19637	Pt_6
Pt_7	407,17		2,99%	18365	16857	375,52	187,76	3,17%	19634	Pt_7
Pt_8	515,09		3,59%	18922	17692	298,69	149,345	3,49%	19469	Pt_8
Pt_9	589,68		3,30%	19328	18522	267,52	133,76	3.46%	19299	Pt_9
Pt_10	443,11		2,78%	19558	18223	355,16	177,58	3,17%	19106	Pt_10
Pt_11	118,57		5,23%	19854	17341	404,93	202,465	2.90%	19657	Pt_11
Mean	379,4		3,84%	19335	17874	348,1	174,0	3,21%	19382	Mean
SD	216,5		1,05%	435	477	65,6	32,8	0,23%	255	SD
Min	97,7		2,78%	18365	16857	267,5	133,8	2,92%	18978	Min
Max	671,6		5,63%	19869	18522	466,2	233,1	3,49%	19657	Max
CTRL_1	266,27		3,64%	19538	16646	419,17	209,585	2,95%	19392	CTRL_1
CTRL_2	167,13		4,52%	19236	16894	413,03	206,515	2,94%	19485	CTRL_2
CTRL_3	370,36		3,15%	17999	16558	655,61	327,805	2.40%	19375	CTRL_3
CTRL_4	267,78		3,52%	19002	17705	417,48	208,74	2,95%	19585	CTRL_4
CTRL_5	275,7		3,42%	19548	18274	337,11	168,555	3.20%	18711	CTRL_5
CTRL_6	500,25		2,64%	19106	18286	563,12	281,56	2,74%	18898	CTRL_6
CTRL_7	1069,8		2,09%	19321	18292	921,97	460,985	1.93%	19182	CTRL_7
CTRL_8	351,01		3,04%	19763	18657	361,22	180,61	3,03%	19723	CTRL_8
CTRL_9	342,27		3,09%	19199	18431	559,22	279,61	2,59%	19737	CTRL_9

CTRL_10	400,88		2,86%	19538	18800	437,28	218,64	3,61%	18554	CTRL_10
CTRL_11	361,61		3,18%	18013	15916	310,84	155,42	3,26%	19549	CTRL_11
Mean	397,6		3,20%	19115	17678	490,6	245,3	3,01%	19290	Mean
SD	239,1		0,62%	592	996	177,5	88,8	0,31%	405	SD
Min	167,1		2,09%	17999	15916	310,8	155,4	2,59%	18554	Min
Max	1069,8		4,52%	19763	18800	922,0	461,0	3,61%	19737	18374

Sample	UROS [5 ng/µL]					UROD [2,5 ng/µL]				
	Raw C/µL	DF (2)	Precision	DP	DP above QT	Raw C/µL	DF (0)	Precision	DP	DP above QT
Pt_1	780,48	390,24	2,24%	19482	18326	295,77		3,37%	19178	17712
Pt_2	887,21	443,605	2,19%	18706	17548	235,78		3,92%	18879	17027
Pt_3	959,09	479,545	2,11%	19117	18101	328,78		3,17%	19182	17156
Pt_4	273,87	136,935	3,50%	18433	17525	180,82		5,51%	19332	18194
Pt_5	391,55	195,775	2,95%	18811	18052	141,34		4,71%	19357	17508
Pt_6	424,08	212,04	2,84%	19387	18146	144,87		4,60%	19570	17797
Pt_7	1086,7	543,35	2,07%	19606	17756	325,24		2,54%	19455	18564
Pt_8	616,06	308,03	2,52%	18850	17180	257,36		3,54%	19796	18473
Pt_9	476,87	238,435	2,81%	18750	16913	234,25		3,76%	19491	18346
Pt_10	626,97	313,485	2,48%	18864	17496	198,57		3,98%	19137	16774
Pt_11	353,64	176,82	3,16%	18607	17288	124,61		5,01%	19342	18204
Mean	625,1	312,6	2,62%	18965	17666	224,3		4,01%	19338	17796
SD	270,5	135,2	0,47%	381	450	73,0		0,88%	245	617
Min	273,9	136,9	2,07%	18433	16913	124,6		2,54%	18879	16774
Max	1086,7	543,4	3,50%	19606	18326	328,8		5,51%	19796	18564
CTRL_1	639,09	319,545	2,51%	19420	16964	403,26		2,90%	19050	17180
CTRL_2	400,17	200,085	3,17%	16843	15309	193,24		4,16%	18902	17159
CTRL_3	618,68	309,34	2,55%	18460	16836	65,034		7,03%	19417	17554
CTRL_4	555,35	277,675	2,59%	19530	17725	168,03		4,44%	19187	17200
CTRL_5	550,44	275,22	2,56%	19482	18090	294,5		3,64%	19226	17777
CTRL_6	761,91	380,955	2,32%	18808	17345	432,94		2,84%	19188	17891
CTRL_7	1252,2	626,1	1,99%	19376	17829	685,19		2,38%	19367	17760
CTRL_8	868,08	434,04	2,20%	19289	17648	256,17		3,67%	19258	16524
CTRL_9	788,23	394,115	2,25%	19325	18022	364,73		3,10%	19179	17410
CTRL_10	854,82	427,41	2,27%	19395	16951	478,55		3,05%	19576	18049
CTRL_11	924,44	462,22	2,29%	17513	15764	191,48		4,36%	19181	18403
Mean	746,7	373,3	2,43%	18858	17135	321,2		3,78%	19230	17537
SD	232,1	116,0	0,31%	903	906	174,8		1,27%	180	517
Min	400,2	200,1	1,99%	16843	15309	65,0		2,38%	18902	16524
Max	1252,2	626,1	3,17%	19530	18090	685,2		7,03%	19576	18403

Sample	PPOX [2,5 ng/µL]					CPOX [5 ng/µL]				
	Raw C/µL	DF (0)	Precision	DP	DP above QT	Raw C/µL	DF (2)	Precision	DP	DP above QT

Pt_1	461,26		2,76%	19178	17712	496,19	248,095	2,65%	19482	18326
Pt_2	396,55		3,11%	18879	17027	638,26	319,13	2,46%	18706	17548
Pt_3	499,05		2,66%	19182	17156	640,79	320,395	2,41%	19117	18101
Pt_4	195,91		5,00%	19332	18194	142,28	71,14	4,76%	18433	17525
Pt_5	175,52		4,25%	19357	17508	251,86	125,93	3,59%	18811	18052
Pt_6	164,82		4,32%	19570	17797	155,54	77,77	4,48%	19387	18146
Pt_7	441,24		2,32%	19455	18564	640,22	320,11	2,24%	19606	17756
Pt_8	335,54		3,14%	19796	18473	249,54	124,77	3,69%	18850	17180
Pt_9	364,47		3,09%	19491	18346	273,44	136,72	3,57%	18750	16913
Pt_10	263,11		3,50%	19137	16774	577,97	288,985	2,56%	18864	17496
Pt_11	147,97		4,61%	19342	18204	144,99	72,495	4,77%	18607	17288
Mean	313,2		3,52%	19338	17796	382,8	191,4	3,38%	18965	17666
SD	129,4		0,88%	245	617	214,9	107,5	0,98%	381	450
Min	148,0		2,32%	18879	16774	142,3	71,1	2,24%	18433	16913
Max	499,1		5,00%	19796	18564	640,8	320,4	4,77%	19606	18326
CTRL_1	542,35		2,57%	19050	17180	620,66	310,33	2,53%	19420	16964
CTRL_2	160,36		4,54%	18902	17159	256,39	128,195	3,86%	16843	15309
CTRL_3	442,5		2,85%	19417	17554	495,25	247,625	2,77%	18460	16836
CTRL_4	170,93		4,40%	19187	17200	365,56	182,78	3,07%	19530	17725
CTRL_5	460,35		3,00%	19226	17777	373,26	186,63	3,00%	19482	18090
CTRL_6	856,43		2,21%	19188	17891	681,16	340,58	2,42%	18808	17345
CTRL_7	1228,2		2,00%	19367	17760	832,21	416,105	2,23%	19376	17829
CTRL_8	490,99		2,77%	19258	16524	470,89	235,445	2,76%	19289	17648
CTRL_9	621,57		2,50%	19179	17410	565,28	282,64	2,54%	19325	18022
CTRL_10	538,4		2,91%	19576	18049	578,75	289,375	2,61%	19395	16951
CTRL_11	383,55		3,18%	19181	18403	601,97	300,985	2,66%	17513	15764
Mean	536,0		2,99%	19230	17537	531,0	265,5	2,77%	18858	17135
SD	300,5		0,81%	180	517	162,0	81,0	0,44%	903	906
Min	160,4		2,00%	18902	16524	256,4	128,2	2,23%	16843	15309
Max	1228,2		4,54%	19576	18403	832,2	416,1	3,86%	19530	18090

Sample	FECH [2,5 ng/µL]					GUSb [0,70 ng/µL]				
	Raw C/µL	DF (0)	Precision	DP	DP above QT	Raw C/µL	MF (3,75)	Precision	DP	DP above QT
Pt_1	1241,3		2,46%	19177	17847	126,68	452,4	4,98%	19071	17882
Pt_2	786,73		2,93%	19477	18238	201,52	719,7	3,94%	19381	18441
Pt_3	823,23		2,24%	19096	17608	210,64	752,3	3,95%	19437	18233
Pt_4	584,44		2,52%	19570	17979	177,998	635,7	6,47%	19336	16617
Pt_5	928,68		2,12%	19465	18239	136,258	486,6	7,76%	19238	18344
Pt_6	987,47		2,09%	19869	18070	186,95	667,7	5,96%	18533	17526
Pt_7	243,11		3,76%	18365	16857	303,63	1084,4	3,37%	18303	17089
Pt_8	846,78		2,90%	18922	17692	132,34	472,6	4,49%	18630	17774
Pt_9	825,91		2,85%	19328	18522	113,33	404,8	4,76%	19130	18075
Pt_10	908,48		2,14%	19558	18223	82,158	293,4	5,15%	19083	17312

Pt_11	866,6		2,23%	19854	17341	57,193	204,3	7,30%	19175	17752
Mean	822,1		2,57%	19335	17874	157,2	561,3	5,28%	19029	17731
SD	248,5		0,51%	435	477	68,6	245,2	1,43%	374	561
Min	243,1		2,09%	18365	16857	57,2	204,3	3,37%	18303	16617
Max	1241,3		3,76%	19869	18522	303,6	1084,4	7,76%	19437	18441
CTRL_1	1452,7		1,99%	19538	16646	222,11	793,3	3,77%	18879	16815
CTRL_2	2454		1,94%	19236	16894	107,83	385,1	5,48%	18630	17700
CTRL_3	2198,4		1,93%	17999	16558	397,76	1420,6	2,51%	18767	17542
CTRL_4	1454,8		1,95%	19002	17705	173,3	618,9	4,27%	18702	17974
CTRL_5	756,32		2,27%	19548	18274	128,23	458,0	4,96%	18520	17857
CTRL_6	1124,3		2,01%	19106	18286	166,61	595,0	3,61%	19513	18453
CTRL_7	3059,9		1,98%	19321	18292	345,37	1233,5	2,84%	18581	17487
CTRL_8	953,84		2,08%	19763	18657	224,35	801,3	3,79%	18685	17980
CTRL_9	1130,6		2,01%	19199	18431	245,18	875,6	3,58%	19376	18494
CTRL_10	1082,9		2,00%	19538	18800	220,45	787,3	3,79%	19237	18200
CTRL_11	1308,8		2,04%	18013	15916	157,28	561,7	4,44%	19308	18252
Mean	1543,3		2,02%	19115	17678	217,1	775,5	3,91%	18927	17887
SD	717,9		0,09%	592	996	88,2	315,1	0,86%	360	490
Min	756,3		1,93%	17999	15916	107,8	385,1	2,51%	18520	16815
Max	3059,9		2,27%	19763	18800	397,8	1420,6	5,48%	19513	18494

Supplementary Table 1: In the first column are shown the samples and the mean; SD, Min and Max. For all genes the following information is reported: The raw data of dPCR experiments expressed in Copies/µL. The raw data adjusted for DF (dilution factor) or MF (multiplication factor) used to consider a total of 2.5 ng of mRNA for all experiment. The precision, the DP (data point) and DP above QT.

EPP_ALAS2 VS	HMBS	ALAD	UROS	UROD	CPOX	PPOX
r	0,40	-0,01	0,03	-0,05	0,18	-0,01
P (two-tailed)	0,22	0,98	0,93	0,88	0,59	0,98
P value summary	ns	ns	ns	ns	ns	ns
CTRL_ALAS2 VS						
r	0,34	-0,15	-0,35	-0,38	-0,14	-0,20
P (two-tailed)	0,31	0,67	0,29	0,25	0,68	0,56
P value summary	ns	ns	ns	ns	ns	ns

Supplementary Table 2: Correlation between ALAS2 vs other genes in EPP patients and CTRL.

3 Supplementary Figures

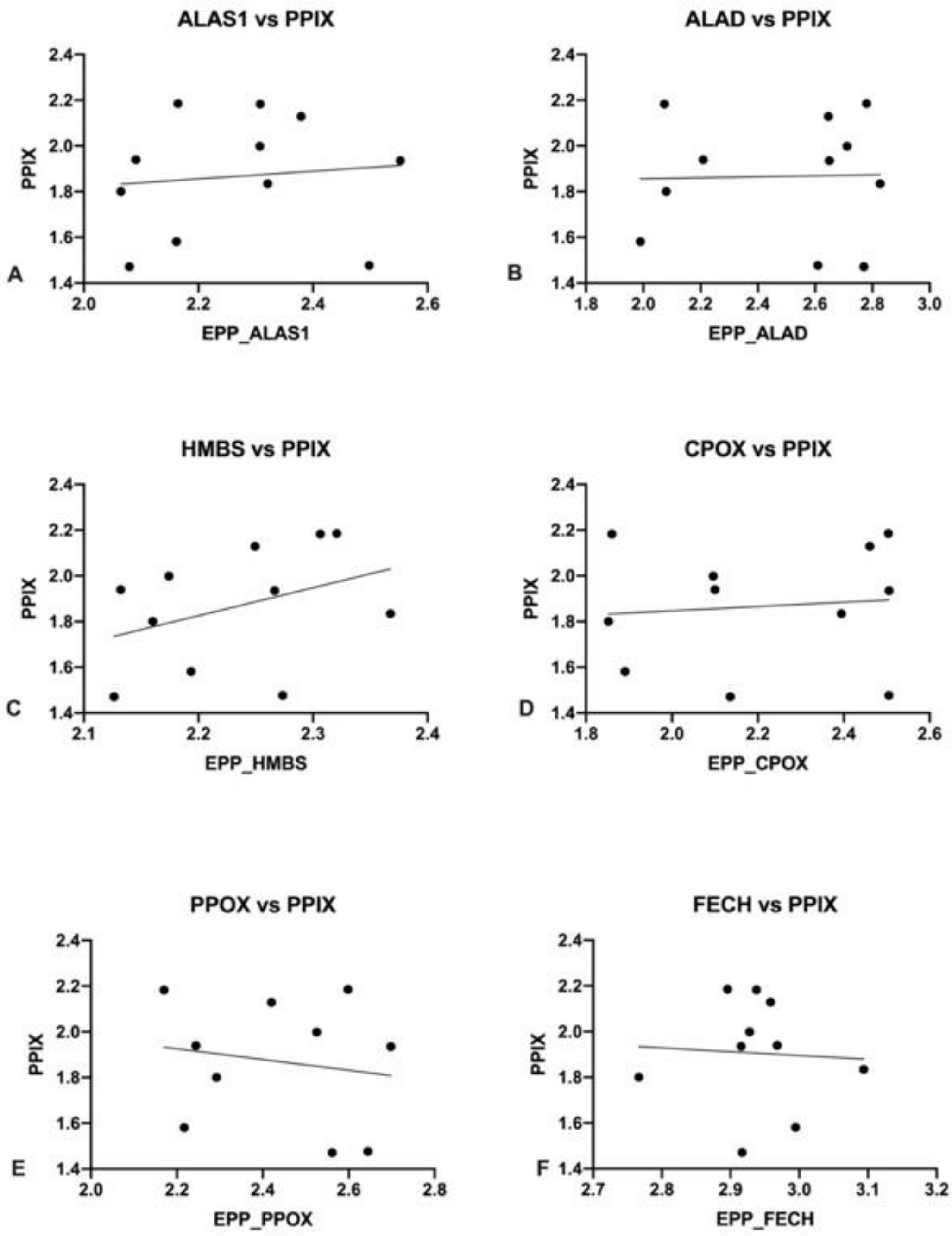

Supplementary Figure 1. Correlation between heme gene expression and PPIX after identification of outlier by ROUT test and normalization. (A) ALAS1 (r=0,1 p= 0,7) (B) ALAD (r=0,02 p= 0,9) (C) HMBS (r=0,3 p= 0,2) (D) CPOX (r=0,09 p= 0,8); (E) PPOX (r= -0,1 p= 0,6); (F) FECH (r= -0.05 p= 0,87).

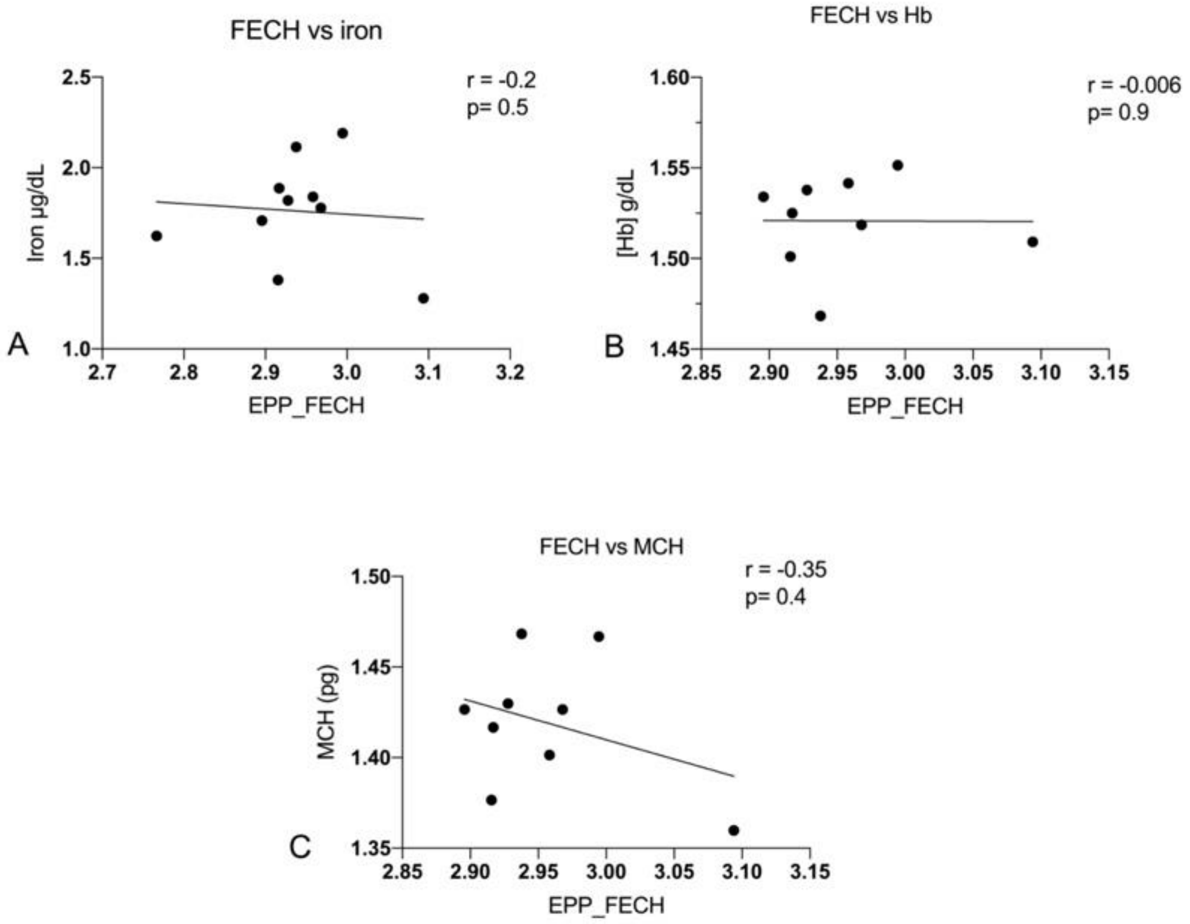

Supplementary Figure 2. (A) Correlation between FECH gene expression and iron (r= - 0,2 p= 0,5) (B) Correlation between FECH gene expression and Hb (r=-0,006 p=0,9) (C). Correlation between FECH gene expression and MCH (r= - 0,35 p=0,4).

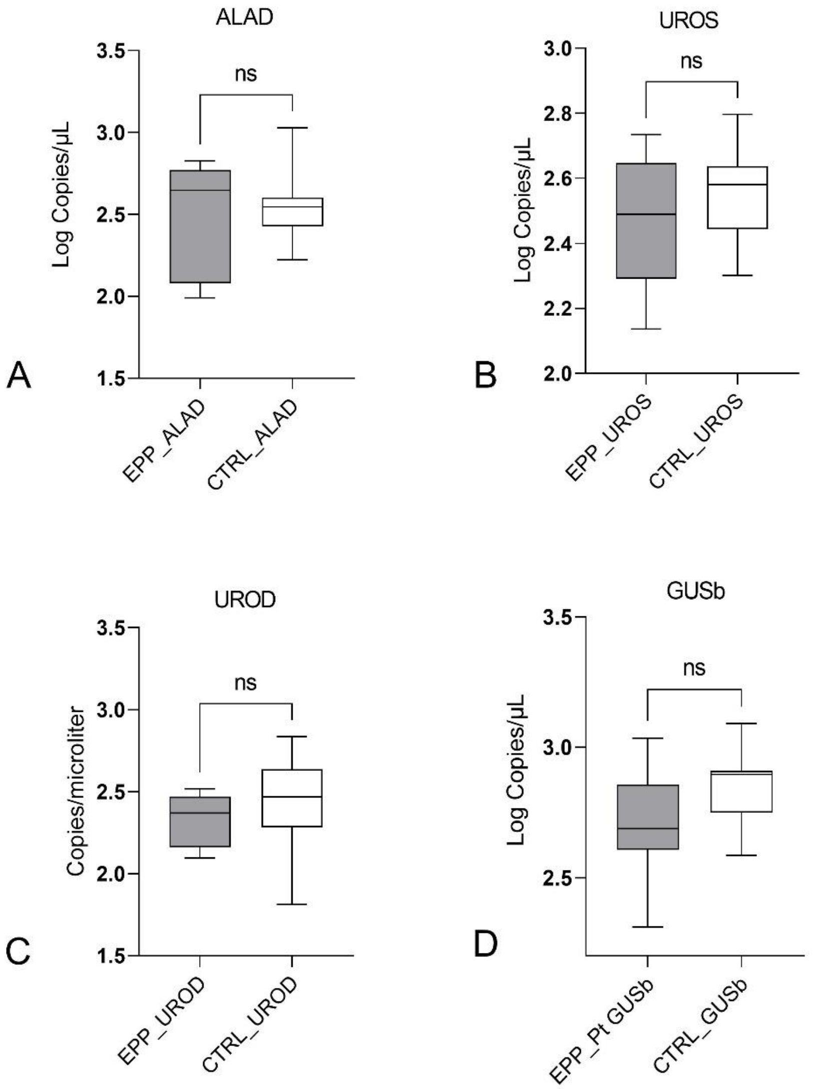

Figure 3: Normal gene expression reported in Log of copies/µl between EPP and CTRL for (A) ALAD; (B) UROS; (C) UROD (D) GUSb. (ns=not significant)

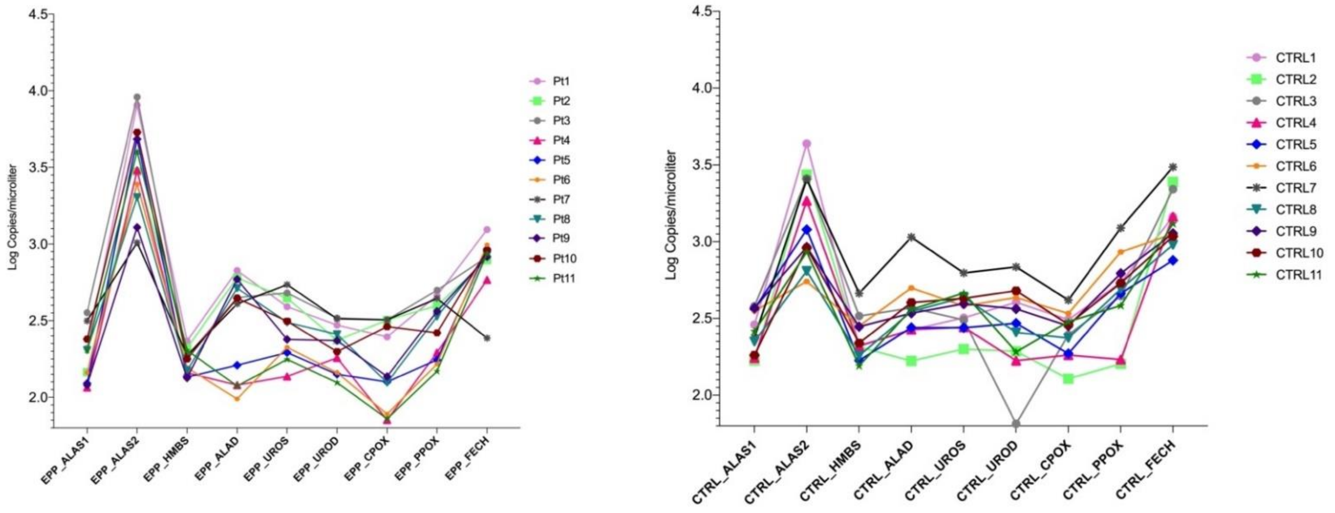

Supplementary Figure 4. Raw data of trend of heme biosynthesis genes expression for patients and controls

4 Others *FECH* mutations

Based on our previous work, by Brancaleoni et al. in 2018 cited in the manuscript, we did not find any difference in the transcription quantification of *FECH* gene in null mutations (including also deletions), by digital PCR, with the same experimental condition (2.5 ng of mRNA).
For this project, we selected the biggest group of patients referring to our center with the same mutation as reported in the manuscript to minimize any unknown genetic variability.
Here we report in supplementary table 3 our previous data from the article on the *FECH* gene; we analyzed 9 different mutations matched with the EPP patients assayed in the present work. Supplementary figure 5 shows no differences between our group with c.[215dupT];[315-48T>C] mutation and the other mutations (the p-value are reported in the figure). At the same time, as expected, we found a difference between the patient's group and the controls.

FECH mut	Raw C/µL	Precision	DP	DP above QT
c.[67+5G>A];[[315-48T>C]	824,37	2,29%	17190	17859
c.[67+5G>A];[[315-48T>C]	662.1	2.55%	15824	18209
c.[343C>T];[315-48T>C]	1021,4	2,10%	17103	18065
c.[706-3C>G];[315-48T>C]	680,78	2,40%	17871	18770
c.[706-3C>G];[315-48T>C]	518,79	2,66%	17021	18698
c.[901_902delTG];[315-48T>C]	489,83	2,78%	16838	18040
c.[801G>A];[315-48T>C]	1039,3	2,12%	17135	18312
Prom-Del3+4	424,15	2,89%	17617	18898
Prom-Del3+4	593,19	2,56%	17113	18629
c.[757_761delAGAAG];[315-48T>C]	417,1	2,88%	17533	19038
c.[757_761delAGAAG];[315-48T>C]	263,6	3,61%	17426	18737
c.[1232G>A];[315-48T>C]	1517	1,98%	16464	18167

Supplementary Table 3: In the first column are shown the *FECH* mutations. The raw data of dPCR experiments expressed in Copies/µL are reported in column 2. The precision, DP (data point) and DP above QT are reported in others columns.

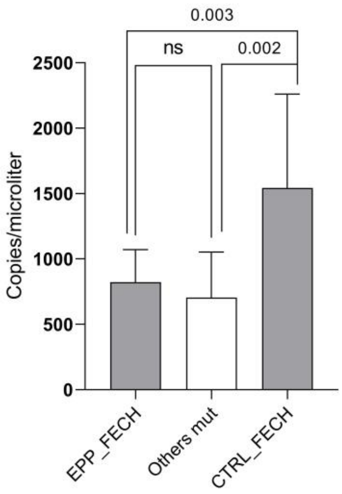

Supplementary Figure 5 Gene expression reported in copies/µl between EPP_FECH (c.215dupT) and EPP with Others mutation ($p=0.4$) (ns=not significant); EPP_FECH vs CTRL_EPP ($p<0.003$); Others mutation vs CTRL_EPP ($p<0.002$).
